# Differential chlorophyll and carotenoid degradation underpin poor curing colour of low-quality flue-cured tobacco leaves

**DOI:** 10.3389/fpls.2026.1745511

**Published:** 2026-01-30

**Authors:** Shikang Fan, Weinan Huang, Yunfei Ma, Yi Chen, Yonglei Jiang, Nan Shi, Dongfang Zheng, Baole Li, Wanpeng Xi, Binbin Hu

**Affiliations:** 1 Yunnan Academy of Tobacco Agricultural Sciences, Kunming, China; 2 College of Horticulture and Landscape Architecture, Southwest University, Chongqing, China

**Keywords:** curing characteristics, flue-cured tobacco, leaf maturity grades, metabolism, quality evaluation

## Abstract

This study investigates the physiological basis for the inferior curing quality of low-quality flue-cured tobacco leaves. Using ‘Yunyan 300’ tobacco, we compared normal (CK), multi-fertilizer (LH), and reviving (FQ) leaves. Post-curing, LH and FQ leaves exhibited uneven, discoloured appearances versus the uniform golden-yellow CK. Physiologically, LH showed delayed water loss and electrolyte leakage, while FQ exhibited premature stress responses. Chemically, low-quality leaves maintained significantly higher post-curing nitrogenous compounds (total nitrogen, alkaloids, protein) and aberrant starch metabolism. Transmission electron microscopy revealed delayed chloroplast disassembly in LH and FQ, with persistent starch granules and tightly packed thylakoids. Pigment analysis showed significantly impaired degradation of chlorophylls and carotenoids in LH and FQ, leading to 30-40% greater accumulation of intermediate catabolites like pheophytin a and pheophorbide a (p < 0.05). Concurrently, the generation of final aroma-active degradation products (e.g., neophytadiene) was reduced. We conclude that the elevated initial levels of chlorophylls and carotenoids, coupled with metabolic dysregulation in low-quality tobacco leaves, collectively impair the complete enzymatic degradation of these pigments. This results in the accumulation of intermediate catabolites and an insufficient generation of key aroma precursors, thereby ultimately leading to the deterioration of both visual appearance and flavor quality.

## 1 Introduction

Tobacco (Nicotiana tabacum L.) is a globally important cash crop, and the quality of its leaves directly determines the sensory properties and market value of cigarette products. Tobacco farming is highly dependent on natural environmental conditions. Even when using advanced cultivation techniques, abnormal or suboptimal leaves can still form in unusual weather conditions (Zhang et al., 2025). In recent years, there have been more frequent extreme weather events during the growth cycle of tobacco plants. This has resulted in the leaves developing a variety of unusual qualities. In recent years, extreme weather events have become more frequent during the growth cycle of tobacco plants. This has resulted in the leaves developing unusual qualities. This severely limits the economic returns of tobacco production. Therefore, it is important to investigate the physiological changes and differences in pigment metabolism that occur when curing tobacco leaves of different qualities.

Conventional chemical metrics remain crucial for evaluating tobacco quality (Feng et al., 2023). Studies indicate that total soluble sugars are the main determinant of sweetness and flavour. Meanwhile, total nitrogen and nicotine levels reflect the intensity of combustion and smoke density (Roemer et al., 2012; Talhout et al., 2006; Tricker et al., 1991). Xiao-Dan et al. (2012) analysed variations in neutral aroma components across five tobacco types. This revealed significant compositional differences that shape aromatic profiles and smoking quality (Severson et al., 1984). In addition to traditional chemical metrics, plastid pigments play a key role in determining leaf appearance and can be precursors to aroma compounds. Their degradation products of these substances constitute 85–96% of the neutral volatile aromas in tobacco (Zhou JiHeng et al., 2004). Notably, neophytadiene, a chlorophyll-derived compound, accounts for over 85% of the volatile aroma components and plays a key role in the aroma differences between varieties (Wu et al., 2024). Neophytadiene reduces smoke irritation and enhances smoothness, and recent studies have linked its abundance to fresh aroma characteristics (Jiheng et al., 2005; Meng et al., 2022). Another key terpenoid class, carotenoids, degrade into around 100 aroma compounds during the curing process (Johnstone and Plimmer, 1959; Meng et al., 2024).

Most Pigment degradation products have low thresholds, minimal irritation, and high aromatic impact, contributing to the refined, elegant, and fresh notes characteristic of flue-cured tobacco. Pigment degradation during curing is influenced by varietal traits, growth environment, maturity, and curing conditions. High humidity and temperature disrupt cellular ultrastructure during curing, enabling enzymatic and oxidative degradation of pigments (Leffingwell, 1999). Although the chlorophyll biosynthetic pathway has been extensively studied and the enzymatic steps are well understood, subsequent metabolic processes remain a significant area of uncertainty. In particular, the fate of chlorophyll and the recycling of its resources during degradation are unclear. While it is known that degradation leads to non-fluorescent chlorophyll catabolites (NCCs), fundamental questions remain regarding their ultimate fate, such as whether they are cleaved into smaller molecules, like monopyrroles, and whether degraded chlorophyll can be used by the plant as a source of nitrogen (Hendry et al., 2010). During the flue-curing process of tobacco, neophytadiene is a key product of chlorophyll degradation and a core component of the neutral aroma constituents in tobacco leaves (Qi et al., 2004). Key enzymes and genes involved in carotenoid synthesis have been isolated. This has enabled genetic modulation of carotenoid content (Sun et al., 2018; Tian et al., 2022). However, oxidative degradation mechanisms remain unclear. Lipoxygenases (chloroplast-derived) and peroxidases (mitochondrial) mediate carotenoid breakdown, both requiring oxygen and cofactors (Nisar et al., 2015). Regional ecology and cultivation practices drive pigment degradation variations. Comparative analyses of flue-cured tobacco from Dali (China), Zimbabwe, and Brazil revealed higher pigment-derived aroma compounds in Dali and Zimbabwean samples, with Yunnan tobacco exhibiting elevated carotenoid degradation products (Hongqi and Jiheng, 2004). Balanced pigment degradation is essential for optimal aroma quality.

Distinct leaf phenotypes, such as multi-fertilizer (LH) and reviving (FQ) leaves, are produced by field practices, climate, and harvest timing (Xie et al., 2017). These low-quality leaves differ significantly in terms of their chemical composition from normal, mature leaves (CK), which impairs their suitability for processing. There is limited research on disparities in chemical and pigment metabolism among leaf phenotypes. This study uses the flue-cured cultivar ‘Yun 300’ to compare the leaves of the LH, FQ and CK varieties under controlled nitrogen regimes. The aim of our findings is to optimise the curing process for low-quality tobacco.

While the pigment metabolism pathways in normally maturing flue-cured tobacco have been extensively studied, a significant knowledge gap remains concerning leaves with inherent quality defects. Previous research has primarily focused on optimal leaves under standard curing regimes. The specific bottlenecks in chlorophyll and carotenoid degradation—particularly relating to chloroplast disassembly and the fate of intermediate catabolites—in low-quality leaves such as LH and FQ subjected to identical curing processes are poorly understood. Elucidating these differential metabolic responses is essential for developing targeted curing strategies to mitigate quality losses.

## 2 Materials and methods

### 2.1 Plant materials

This study was conducted in Chengjiang City, Yuxi Prefecture, Yunnan Province (23 °19′ to 24 °53′ N, 101 °16′ to 103 °09′ E) during the 2023 growing season. The experimental site features a subtropical plateau monsoon climate, characterized by an annual mean temperature range of 16.4 - 24.6°C. The region exhibits mild winters without severe cold and temperate summers without extreme heat, maintaining spring-like conditions year-round with distinct wet and dry seasons. The trial utilized the locally cultivated tobacco variety Yunyan 300, with key phenological stages occurring on April 5 (transplanting) and July 9 (topping). The field trial employed a randomized complete block design with three independent biological replicates. Each replicate plot consisted of 80 plants, with a row spacing of 1.2 m and a plant spacing of 0.5 m. Three experimental treatments were established based on regional fertilization practices: control group (standard fertilization protocol),”Multi fertilizer” treatment and “Reviving” treatment. Detailed fertilization parameters for each treatment group are systematically presented in **Table 1**.

**Table 1:** Record of fertilization.

Tobacco leaves of different qualities	Nitrogen consumption	Base fertilizer 12:6:24 compound fertilizer	Seedling fertilizer 28:0:5	Top application 12:6:24 compound fertilizer	Apply fertilizer 12:6:24 compound fertilizer
Y300-CK	8.25 g/m^2^	18 g/m^2^	7.5 g/m^2^	36.3 g/m^2^	
		May 2	May 12	May 17	
Y300-Multi fertilizer	14.25 g/m^2^	18 g/m^2^	7.5 g/m^2^	61.2 g/m^2^	25.05 g/m^2^
		May 2	May 12	May 17	June 22
Y300-Reviving	14.25 g/m^2^	18 g/m^2^	7.5 g/m^2^	61.2 g/m^2^	25.05 g/m^2^
		May 2	May 12	May 17	July 10

Normal leaves (CK):SPAD value ~28–32 at harvest. Multi-fertilizer leaves (LH) and Reviving leaves (FQ): SPAD value >35 at harvest.

### 2.2 Sampling and testing requirements

**Figure 1:**
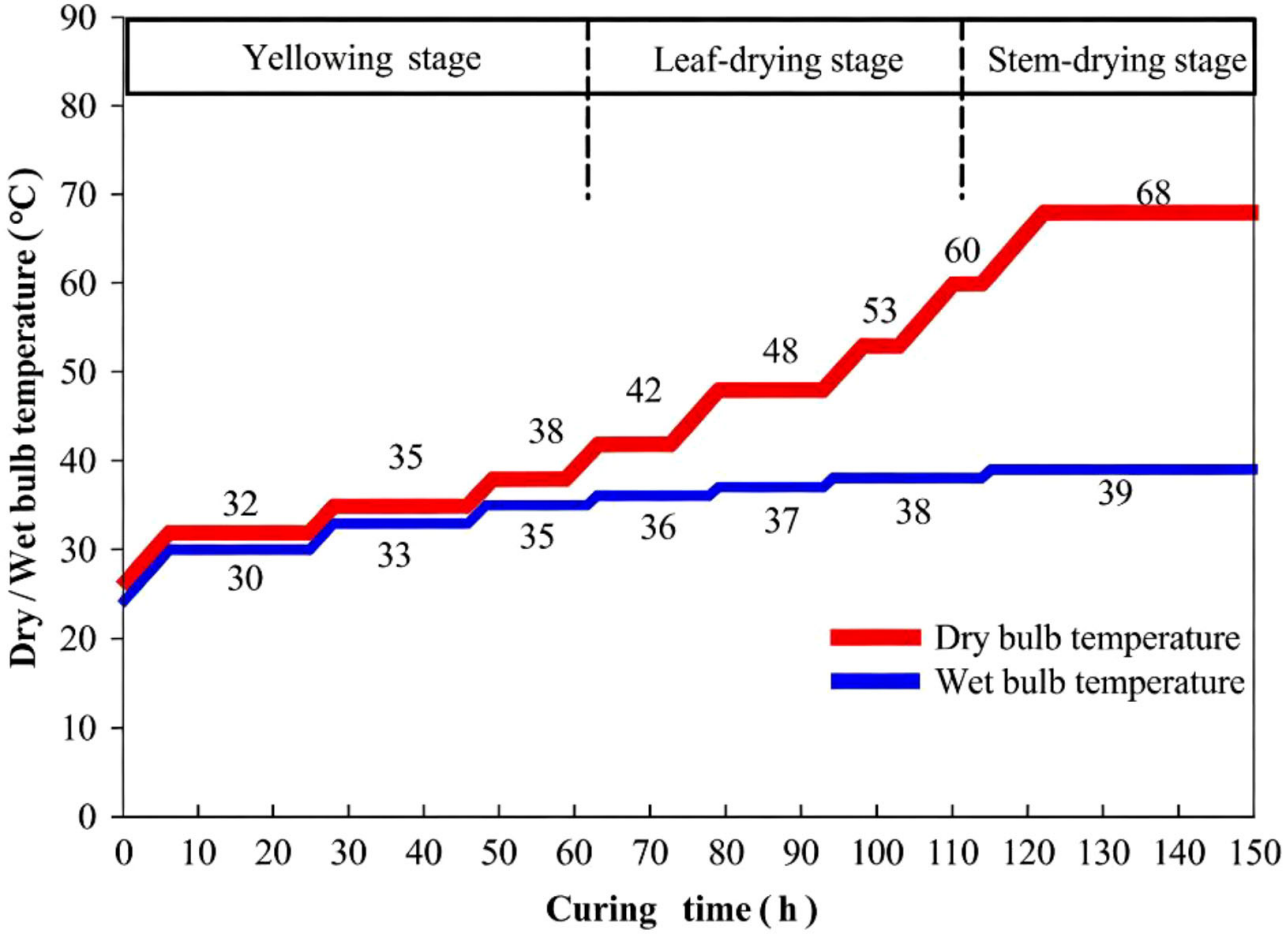
Tobacco flue-curing process.

The Tobacco flue-curing process (illustrated in **Figure 1**) involved sampling at five key stages: green tobacco leaves, post-drying at 38 °C, 42 °C, 48 °C, and cured. Three replicates were collected at each stage. From each replicate, five leaf discs were collected, from which the midrib was excised. Subsequently, 2×2 cm lamina pieces were obtained for analysis. Samples were rapidly frozen in liquid nitrogen.

Then stored at -80°C for subsequent analysis of conventional chemical constituents (including total sugars, reducing sugars, starch, total nitrogen, total alkaloids, protein, and chlorine) as well as Chlorophyll and its related metabolites, Carotenoids and their related metabolites contents in different leaf sections. All frozen samples were protected from light and analyzed within 3 days of storage to minimize pigment degradation. Additionally, for each treatment condition (normal, multi fertilizer, and reviving). Thereafter, a portable colorimeter was employed to assess colorimetric values (L, a, b values) across various leaf sections, followed by histological examination of leaf cell structures post-sectioning. Leaf color was measured using a Konica-Minolta CM-600d spectrophotometer with an 8 mm aperture, calibrated against a standard white tile. Three readings were taken and averaged per leaf sample.

### 2.3 Determination of carbohydrate compound content

#### 2.3.1 Determination of total sugars and reducing sugars

The determination of total sugars and reducing sugars follows the method outlined in “Tobacco and tobacco products─Determination of water soluble sugars─Continuous flow method YC/T159-2019” (Yang et al., 2025). Specifically, 0.25 g of the sample is weighed into a 50 mL stoppered conical flask with precision to 0.0001 g. Subsequently, 25 mL of 5% acetic acid solution is added, and the flask is sealed and agitated on a shaker (at a speed exceeding 150 rpm) for 30 minutes to facilitate extraction. The extracted solution is filtered through rapid qualitative filter paper, with the initial 2–3 mL of filtrate discarded. The subsequent filtrate is collected for analytical purposes.

A series of standard working solutions and the processed filtrate from the sample are analyzed using the instrument. If the sample concentration exceeds the range of the standard working solutions, dilution is required prior to measurement.

#### 2.3.2 Determination of starch

The determination of starch content follows the method outlined in “Tobacco and tobacco products—Determination of starch—Continuous flow method:YC/T216-2013” (Yang et al., 2025). Samples were prepared according to YC/T31, and their moisture content was subsequently measured. A precisely weighed 0.25 g sample was placed into a 50 mL G3 sintered glass funnel. Subsequently, 25 mL of an 80% ethanol-saturated sodium chloride solution was added to the funnel, which was then immersed in a 400 mL beaker containing an appropriate amount of water. Ultrasonic extraction (350 W power) was performed at room temperature for 30 minutes. After extraction, the funnel was removed, and the extraction solution was discarded by opening the stopcock. The sample residue within the funnel was rinsed with 2 mL of the 80% ethanol-saturated sodium chloride solution, and the rinsate was discarded by applying pressure with a double-bulb aspirator before closing the stopcock. The funnel was returned to the 400 mL beaker, and 15 mL of a 40% perchloric acid solution was added to the sample residue. Ultrasonic extraction (350 W power) was conducted at room temperature for 10 minutes. Following this, 15 mL of water was added to the funnel, mixed thoroughly, and the starch extract was collected in a 50 mL Erlenmeyer flask by opening the stopcock. A 5 mL aliquot of the extract was accurately transferred to a 50 mL volumetric flask and diluted to the mark with water, then mixed thoroughly for analysis.

### 2.4 Determination of nitrogenous compound content

#### 2.4.1 Determination of total alkaloid content

The determination of total alkaloid content follows the method outlined in” Tobacco and tobacco products—Determination of total alkaloids—Continuous flow (potassium thiocyanate) method: YC/T468-2021” (Yang et al., 2025), Samples were prepared according to YC/T31, and their moisture content was subsequently measured. A precisely weighed 0.25 g sample (to the nearest 0.0001 g) was placed into a 50 mL conical flask with a stopper. Then, 25 mL of water was added, the stopper was secured, and the flask was shaken on a shaker (at a speed exceeding 150 rpm) for 30 minutes to extract the alkaloids. The extraction solution was filtered using rapid qualitative filter paper, and the initial few milliliters (2 mL–3 mL) of the filtrate were discarded. The subsequent filtrate was collected for analysis.

#### 2.4.2 Determination of protein content

The determination of protein content follows the method outlined in” Tobacco and tobacco products—Determination of protein—Continuous flow method: YC/T249-2008” (Yang et al., 2025), Samples are prepared according to YC/T31, and moisture content is measured. Approximately 0.5 g of the sample is weighed into a 100 mL conical flask, with precision to 0.0001 g. Twenty-five milliliters of acetic acid solution is added, and the mixture is gently heated, maintaining boiling for 15 minutes. The solution is rapidly filtered through quantitative filter paper in a vacuum filtration apparatus, and the conical flask and precipitate are rinsed with acetic acid solution until the filtrate is colorless. The filter paper and precipitate are then transferred to a digestion tube. To the digestion tube, 0.1 g of mercury (II) oxide, 1.0 g of potassium sulfate, and 5 mL of concentrated sulfuric acid are added. The digestion tube is placed on a digestion apparatus for digestion. The following operating parameters for the digestion apparatus are recommended: maintain at 150 °C for 1 hour, then increase the temperature to 370 °C and maintain for 4 hours. After digestion, the tube is allowed to cool slightly, and a small amount of water is added. Once cooled to room temperature, the volume is adjusted to the mark with water, and the solution is thoroughly mixed.

#### 2.4.3 Determination of total nitrogen content

The determination of total nitrogen content follows the method outlined in “Tobacco and tobacco products—Determination of total nitrogen—Continuous flow method: YC/T161-2002” (Yang et al., 2025), Test samples are prepared according to YC/T31, and moisture content is measured. Approximately 0.1 g of the test material is weighed into a digestion tube, with precision to 0.0001 g. To the digestion tube, 0.1 g of mercury (II) oxide, 1.0 g of potassium sulfate, and 5.0 mL of concentrated sulfuric acid are added. The digestion tube is placed on a digestion apparatus for digestion. The operating parameters for the digestion apparatus are as follows: 150 °C for 1 hour, followed by 370 °C for 1 hour. After digestion, the tube is allowed to cool slightly, and a small amount of water is added. Once cooled to room temperature, the volume is adjusted to the mark with water, and the solution is thoroughly mixed.

### 2.5 Determination of mineral element content

#### 2.5.1 Determination of chlorine content

The determination of chlorine content follows the method outlined in “Tobacco and tobacco products—Determination of chloride—Continuous flow method: YC/T162-2011” (Yang et al., 2025), Test samples are prepared according to YC/T31, and their moisture content is measured. Approximately 0.25 g of the test sample is weighed into a 50 mL stoppered conical flask, with precision to 0.1 mg. To this, 25 mL of water is added, the flask is stoppered, and the mixture is extracted for 30 minutes on a shaker at a speed greater than 150 revolutions per minute (rpm). The extraction solution is filtered using rapid qualitative filter paper, discarding the first 2 mL to 3 mL of filtrate, and the subsequent filtrate is collected for analysis.

### 2.6 Determination of metabolites related to pigment metabolism element content

#### 2.6.1 Determination of chlorophyll content

Chlorophyll levels were quantified using high-performance liquid chromatography (HPLC) as per YC/T 382-2010. Accurately weigh 0.2 g of the ground and prepared sample into a 50 mL Erlenmeyer flask, then precisely add 25 mL of the extractant (90% acetone solution). Perform ultrasonic extraction at room temperature for 20 min. Take an appropriate amount of the extract and filter it through a 0.45 μm micro-membrane filter. Transfer the filtrate into a 2 mL brown chromatographic vial for subsequent HPLC analysis. For HPLC analyses, recovery rates for spiked standards ranged from 85–105%. The relative standard deviation (RSD) was <5% for intra-day and <8% for inter-day precision.

The analytical conditions adopted are as follows: The chromatographic column is Waters Nova - Pak - Cis (3.9 × 150 mm, 4 μm); the column temperature is set at 40 °C; the column flow rate is 0.5 mL/min; the injection volume is 1 μL; mobile phase A is isopropanol; mobile phase B is an 80% acetonitrile aqueous solution (V/V); the equilibrium time is 6 min; gradient elution is applied. The detection wavelength is 450 nm. Qualitative analysis is carried out by retention time, and quantitative analysis is performed using the external standard method.

#### 2.6.2 Determination of phaeophorbide A and Py-A content

For Phaeophorbide A, tissue homogenization is performed as follows: Weigh 0.1–0.5 g (minimum 5–10 mg) of tissue blocks, rinse in ice - cold PBS, dry with filter paper, and transfer to a 5-mL homogenization tube. Add 9 volumes of homogenization medium (e.g., 0.05 mol/L Tris-HCl, pH 7.4 PBS) relative to the tissue weight (g: mL=1:9), mince the tissue with ophthalmic scissors in an ice - water bath, and homogenize manually or mechanically. Manual homogenization involves grinding the tissue in an ice - water bath for 6–8 min until a 10% homogenate is formed. Mechanical homogenization uses a tissue grinder (10,000–15,000 rpm) in ice water. The homogenate is centrifuged at 3,000 × g for 10–15 min, and the supernatant is collected. For Py-A, plant extracts or related samples are centrifuged at 1,000 × g for 20 min, and the supernatant is used directly. Both samples should be aliquoted and stored at - 20°C to avoid repeated freeze-thaw cycles, and thawed at room temperature before use. For the ELISA kits, cross-reactivity with related compounds was validated per the manufacturer’s specifications, ensuring specificity for pheophorbide A and Py-A.

The detection principle for both is based on the double - antibody sandwich ELISA. Microplates pre-coated with specific antibodies against Phaeophorbide A or Py-A are used. Standards, samples, and HRP-conjugated detection antibodies are sequentially added, followed by incubation and washing. Substrates TMB (chromogen A and B) are added, and the reaction is stopped by acid, converting the blue product to yellow. The absorbance at 450 nm is measured using a microplate reader, and the sample concentration is calculated from a standard curve (standard concentrations: 0, 20, 40, 80, 160, 320 μg/mL). The operation steps include balancing reagents at room temperature, adding 50 μL standards or 10 μL samples (diluted with 40 μL sample diluent), incubating with HRP-conjugate at 37°C for 60 min, washing 5 times, developing with substrates for 15 min in the dark, and measuring OD within 15 min after adding stop solution. The kit performance features include a linear regression coefficient (R ≥ 0.99), sensitivity < 1.0 μg/mL, and CV < 15% for intra - and inter-plate variations.

#### 2.6.3 Determination of carotenoid content

HPLC grade methanol (MeOH), ethanol (EtOH), and acetonitrile (ACN) were purchased from Merck (Darmstadt, Germany). BHT was purchased from Aladdin. Acetone was purchased from Sinopharm. Methyl tert-butyl ether (MTBE) was purchased from CNW. NaCl was purchased from Rhawn. KOH was purchased from Hushi. MilliQ water (Millipore, Bradford, USA) was used in all experiments. All of the standards were purchased from Sigma-Aldrich (St Louis, MO, USA) and BOC (NY, USA). Formic acid was obtained from Sigma-Aldrich. The stock solutions of standards were prepared at the concentration of 1 mg/mL in MTBE/MeOH. All stock solutions were stored at -20 °C. For LC-MS/MS (APCI+) carotenoid analysis, qualifier/quantifier ion ratios were monitored for all compounds. Analyses were accepted only if the deviation of these ratios from the mean standard ratio was <20%.

The sample was freeze-dried, ground into powder (30 Hz, 1.5 min), and stored at -80 °C until needed. 50 mg powder was weighted and extracted with 0.5 mL mixed solution of n-hexane: acetone: ethanol (1:1:1, v/v/v). The extract was vortexed for 20 min at room temperature. The supernatants were collected after centrifuged at 12000 r/min for 5 min at 4 °C. The residue was re-extracted by repeating the above steps again under the same conditions. And then evaporated to dryness, reconstituted in 100μL dichloromethane. The solution was filtered through a 0.22 μm membrane filter for further LC-MS/MS analysis.

The sample extracts were analyzed using an UPLC-APCI-MS/MS system (UPLC, ExionLC™ AD; MS,Applied Biosystems 6500 Triple Quadrupole). The analytical conditions were as follow, LC: column, YMC C30 (3 μm, 100 mm×2.0 mm i.d); solvent system, methanol:acetonitrile (1:3, v/v) with 0.01% BHT and 0.1% formic acid (A), methyl tert-butyl ether with 0.01% BHT (B); gradient program, started at 0% B (0–3 min), increased to 70% B (3–5 min), then increased to 95% B (5–9 min), finaly ramped back to 0% B (10–11 min); flow rate, 0.8 mL/min; temperature, 28 °C; injection volume: 2 μL.

Linear ion trap (LIT) and triple quadrupole (QQQ) scans were acquired on a triple quadrupole-linear ion trap mass spectrometer (QTRAP), QTRAP^®^ 6500+ LC-MS/MS System, equipped with an APCI Heated Nebulizer, operating in positive ion mode and controlled by Analyst 1.6.3 software (Sciex). The APCI source operation parameters were as follows: ion source, APCI+; source temperature 350 °C; curtain gas (CUR) were set at 25.0 psi. Carotenoids were analyzed using scheduled multiple reaction monitoring (MRM). Data acquisitions were performed using Analyst 1.6.3 software (Sciex). Multiquant 3.0.3 software (Sciex) was used to quantify all metabolites. Mass spectrometer parameters including the declustering potentials (DP) and collision energies (CE) for individual MRM transitions were done with further DP and CE optimization. A specific set of MRM transitions were monitored for each period according to the metabolites eluted within this period.

#### 2.6.4 Determination of pigment degradation products content

Pigment degradation products were analyzed via gas chromatography-mass spectrometry (GC-MS) (Jianmin et al., 2013). Accurately weigh 0.5 g of the sample into a 50 mL round - bottom flask. Subsequently, add 0.4 g of NaCl and 8 mL of deionized water. After shaking well, connect one end of the flask to the Simultaneous Distillation and Extraction (SDE) apparatus. The other end of the apparatus is connected to a flat - bottomed small flask containing 30 mL of dichloromethane. Then, place the end of the flat - bottomed small flask in a 60 °C water bath for heating, and put the end of the large round - bottom flask on an electric heating mantle for heating. After simultaneous distillation and extraction for 3 h, dry the extract with anhydrous sodium sulfate. Then, add 1 mL of 1 - decanol as an internal standard. Concentrate the solution to 1 mL by rotary evaporation in a 40 °C water bath using a rotary evaporator. Pass the concentrated solution through a 0.45 μm filter membrane into a sampling vial for GC/MS analysis.

The gas chromatography - mass spectrometry (GC/MS) analysis was performed using an HP - 5MS column (Agilent 19091S - 433, 30 m × 0.25 mm × 0.25 μm). The inlet temperature was set at 260 °C, and the injection volume was 1 μL with a split ratio of 20:1. The interface temperature was 280 °C. The temperature - programming protocol was as follows: start at 60 °C for 2 min, then ramp up to 280 °C, hold at 280 °C for 10 min, cool down to 260 °C and hold for 10 min, and then increase the temperature at a rate of 5 °C/min (the final target temperature in this stage was not clearly stated in the original text, which may be an error). Helium was used as the carrier gas and the make - up gas, with a flow rate of 1.2 mL/min. The ion source temperature was 230 °C, and the quadrupole temperature was 150 °C. The scanning range was 30–550 aum. The NIST08 library and the Agilent ChemStation were used for data acquisition and analysis.

### 2.7 Data processing

Statistical significance was assessed using Microsoft Excel and GraphPad Prism. Shapiro–Wilk and Levene tests were applied; Means were compared by Tukey HSD (GraphPad Prism 10.0). Differences with P < 0.05 were considered statistically significant.

## 3 Results and analysis

### 3.1 Color changes in tobacco leaves during curing

**Figure 2:**
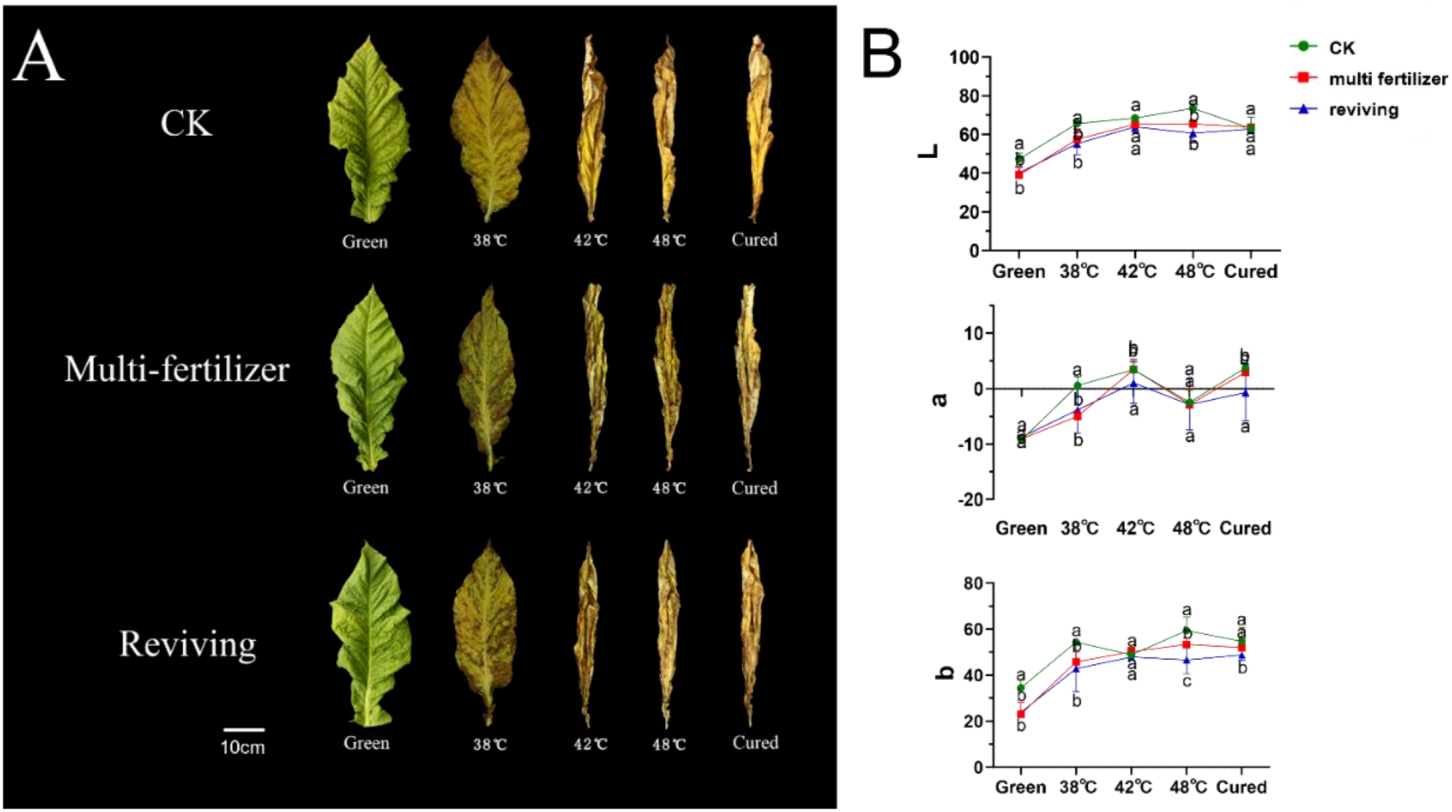
Color changes and Lab values of tobacco leaves with CK,Multi-fertilizer and Reviving groups during curing. Different lowercase letters within the same column indicate significant differences (P < 0.05); the same applies below.

The colour of the leaves is a critical indicator of the quality of the tobacco, with distinct visual differences observed during the curing process for leaves of different initial qualities (Li-Ting et al., 2014). The CK group exhibited a uniform yellow gradient during the curing process, ultimately achieving an even golden-yellow colour. (**Figure 2A**). In contrast, the color of LH group and FQ group showed abnormal changes. The former group displayed delayed and uneven chlorophyll degradation, with pronounced chlorophyll retention in the vein regions, resulting in a dull yellow coloration accompanied by dark brown patches. The latter group exhibit irregular breakdown of chlorophyll in their leaves, with localised areas turning brown or green and withering. Ultimately, this results in yellowish-brown colouration, accompanied by mottled scorching and desiccation.

Furthermore, we conducted a quantitative analysis of the colour changes in tobacco leaves across three groups. The results showed that CK exhibited a typical colour transformation during curing. L* values (lightness) steadily increased, a* values transitioned from negative (green) to positive (red), and b* values (yellow intensity) rose consistently. These coordinated changes resulted in a uniform golden-yellow appearance with stable, harmonious colour development (**Figure 2B**). By contrast, LH exhibited a significantly smaller increase in L* values, slower and weaker shifts towards red tones in a* values, and limited growth in b* values. This resulted in a dull yellow-to-brown colour. FQ displayed distinct behaviour: The L* values decreased intermittently, the a* values fluctuated irregularly between red and green hues, and the b* values consistently remained lower than the averages for CK, producing a patchy yellow-green appearance (see **Figure 2B**).

### 3.3 Tissue stability of tobacco leaves during curing

**Figure 3:**
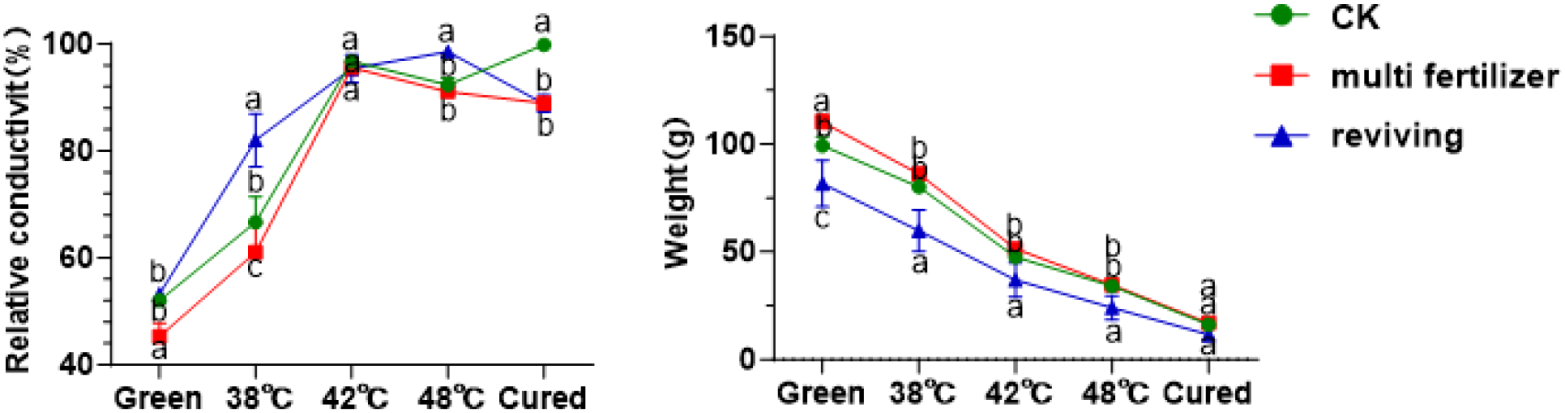
Relative conductivity and weight of tobacco leaves with CK,Multi-fertilizer and Reviving groups during curing process.

Relative electrical conductivity (REC) is a key physiological indicator of the health and integrity of a plant’s cellular membrane systems. It is widely used to evaluate the tolerance of tobacco leaves to curing (**Figure 3**) (Li et al., 2022). The results showed that the REC values for the CK, LH and FQ groups increased steadily during the curing process, reaching a peak and then stabilising at 42 °C. The FQ group exhibited a sharp rise in relative electrical conductivity (REC) from fresh leaves to 38 °C, while the LH and CK groups showed similar surges between 38 °C and 42 °C, with the LH group showing the latest surge. Despite minor differences in total weight loss, the FQ group experienced rapid weight loss from fresh leaves at 38 °C. In contrast, the LH and CK groups displayed abrupt reductions in weight at temperatures between 38 °C and 42 °C; again, the LH group lagged behind (**Figure 3**).

### 3.4 Carbon-nitrogen metabolism and enzyme dynamics

**Figure 4:**
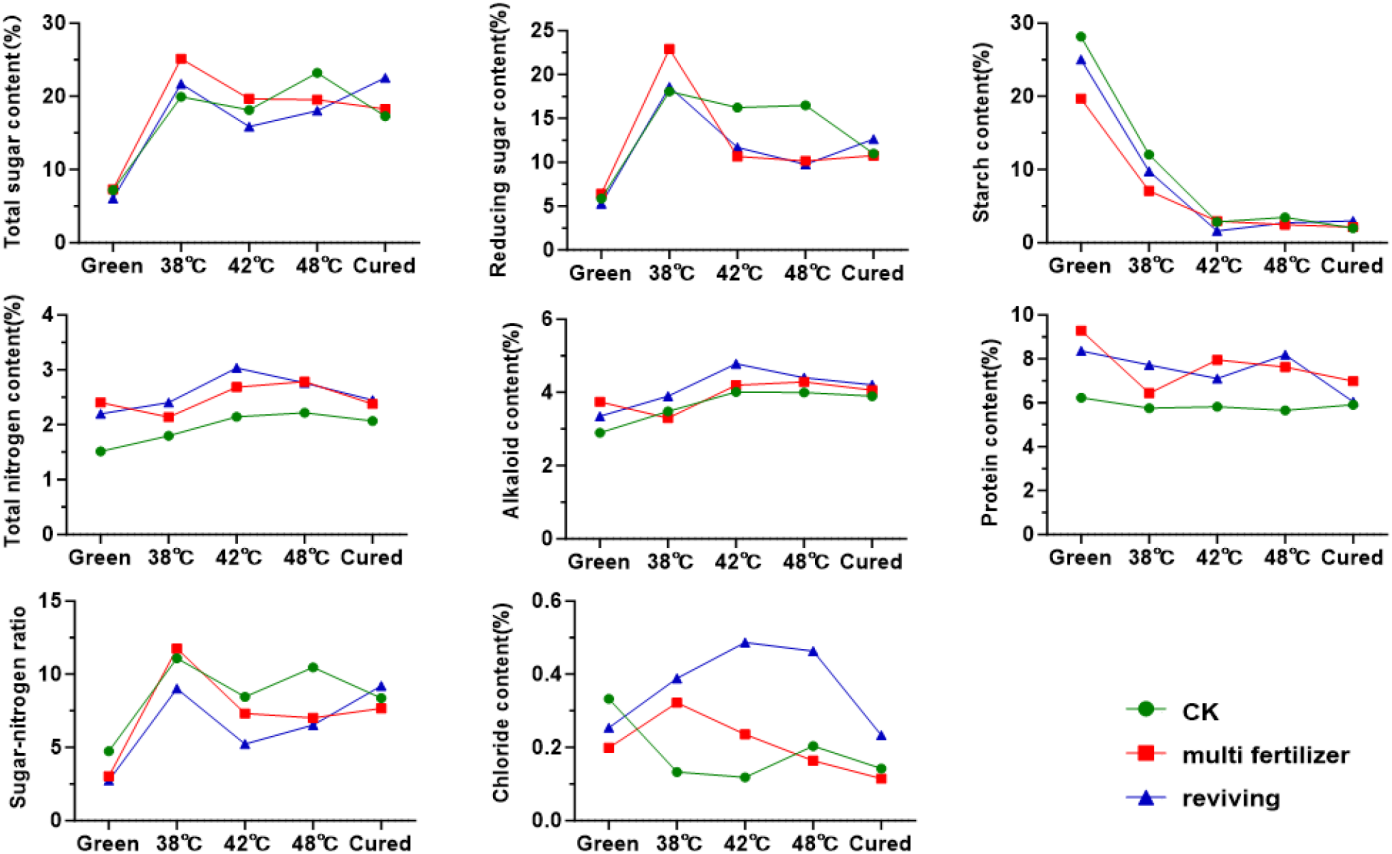
Conventional chemical components of tobacco leaves with CK, Multi-fertilizer, and Reviving groups during curing process.

Overall, starch and sugar metabolism followed opposing trends during the curing process. The starch content of the leaves decreased sharply from fresh to 42 °C, after which it stabilised with no significant differences (**Figure 4**). Meanwhile, the levels of reducing and total sugars rose rapidly to peak at 38 °C, after which they gradually declined. Notably, the LH group initially had higher total and reducing sugars, as well as lower starch, than the CK group, but ended the curing process with 5.5% and 7.5% higher total sugars and starch, respectively, and 2% lower reducing sugars. In contrast, the FQ group started with significantly lower levels of sugars and starch than the CK group, but surpassed them post-curing. Similar patterns were observed in total nitrogen and alkaloid levels in all leaf grades, with a gradual rise up to 42 °C, followed by a slow decline. Conversely, total protein content decreased continuously throughout the curing process. The LH and FQ groups consistently exhibited higher levels of total nitrogen, alkaloids and protein than the CK group, with the LH group ranking highest. The sugar-to-nitrogen ratio mirrored the trend for reducing sugars, peaking early before declining sharply and stabilising after 42 °C. The chloride ion content in the FQ group remained significantly higher than in the CK and LH groups.

**Figure 5:**
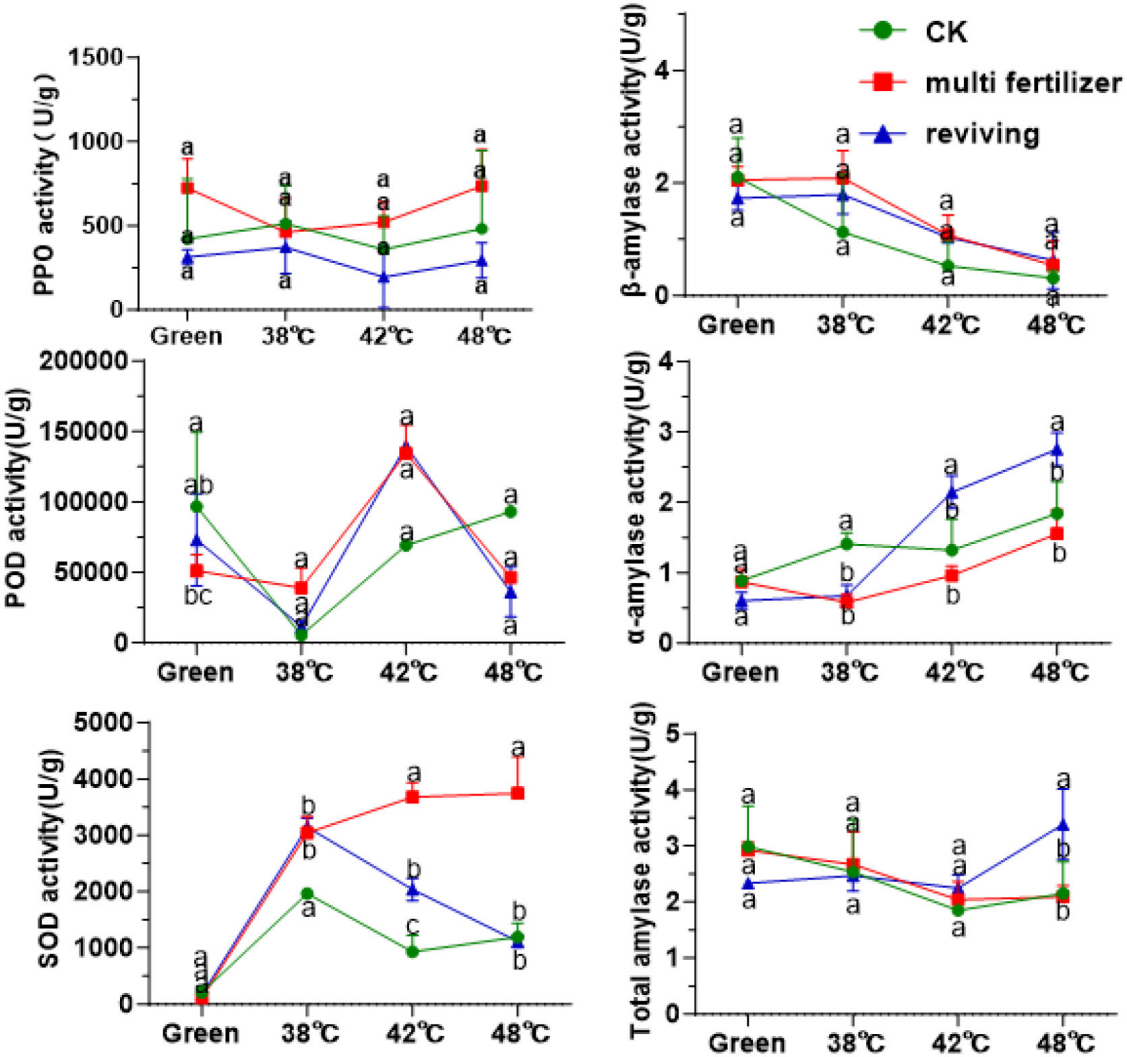
Enzyme activity during curing process of tobacco leaves with CK,Multi-fertilizer, and Reviving groups; One unit (U) of PPO activity is defined as a change of 0.01 in absorbance per minute per gram fresh weight.; One unit (U) of SOD activity is defined as the amount causing 50% inhibition of the photochemical reduction of nitroblue tetrazolium (NBT).

Furthermore, this study measured the activities of enzymes in tobacco leaves of varying quality during the curing process. As illustrated in **Figure 5**, the activity of polyphenol oxidase (PPO) initially decreased and then increased in the CK, LH and FQ groups across the curing stages. Throughout the flue-curing stages, PPO activity remained relatively stable and showed no significant variations. Among the treatment groups, the LH group consistently demonstrated the highest overall PPO activity, followed by the CK group, while the FQ group exhibited the lowest. In contrast, the activities of both peroxidase (POD) and superoxide dismutase (SOD) increased progressively during curing. Similarly, the LH group registered the highest activities of these enzymes, and the CK group the lowest. These enzymatic patterns imply that the LH group, characterized by lower leaf maturity and sustained higher antioxidant enzyme activity, potentially contributed to its enhanced tolerance to thermal stress during the curing process. Analysis of starch metabolism enzymes revealed that β-amylase activity declined steadily during curing in all leaf types, whereas α-amylase activity showed an opposing increase. This dynamic resulted in an initial decrease in total amylase activity, followed by a recovery and stabilization between 2–3 U/g. In terms of chemical component balance, the CK group leaves demonstrated a superior harmonious composition compared to the poorer coordination observed in the LH and FQ groups.

### 3.5 Chlorophyll degradation and chloroplast ultrastructural changes

**Figure 6:**
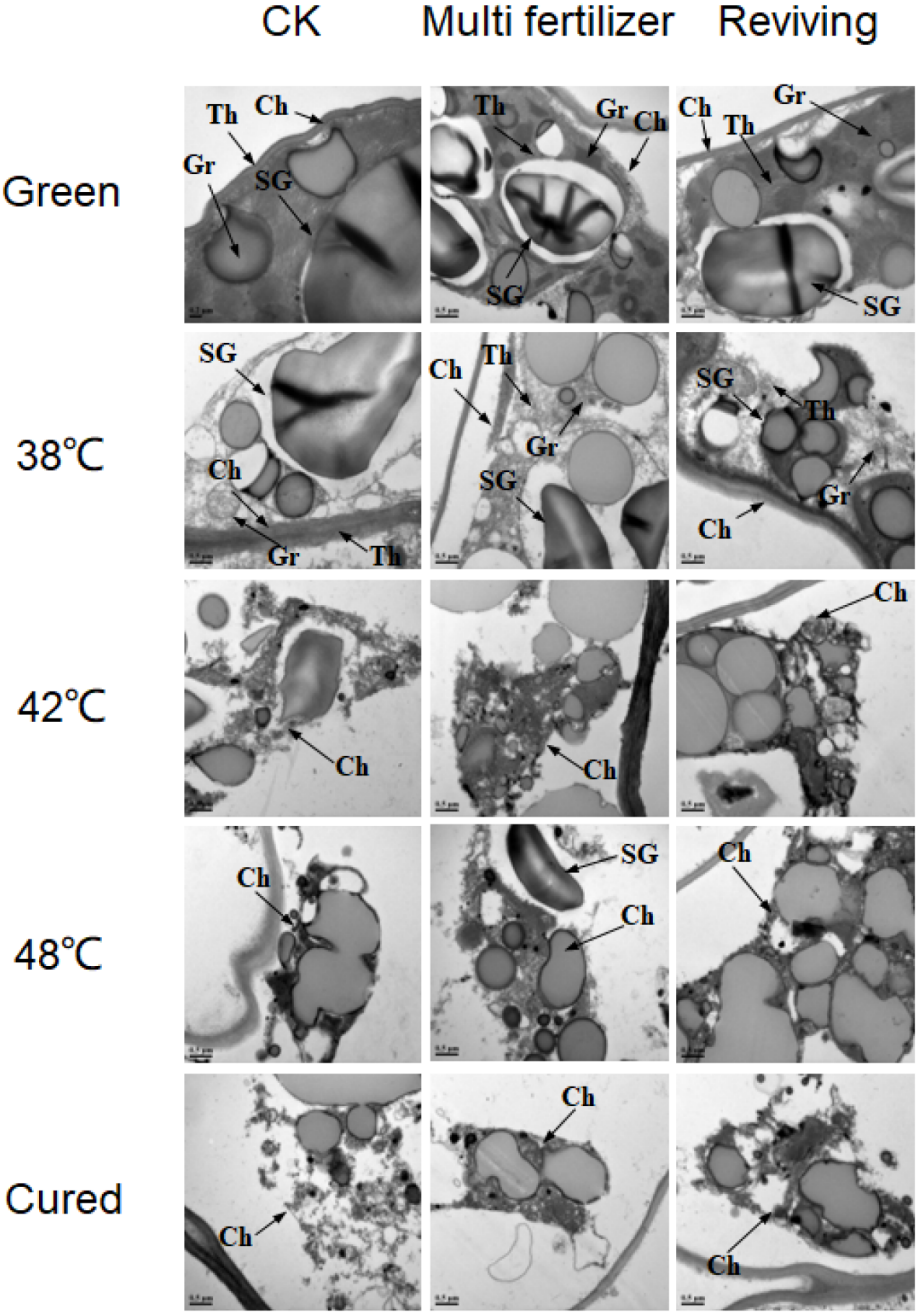
Ultrastructure of chloroplasts in tobacco leaves of CK. Multi-fertilizer and Reviving groups during flue-curing. Ch – Chloroplast; SG - Starch Grain; Gr - Granum Thylakoid; Th - Stroma Thylakoid; scale bar = 0.5 μm; Samples for TEM were taken from the interveinal lamina, specifically from the 2nd to 3rd layer of spongy mesophyll cells.

Ultrastructural analysis via electron microscopy further elucidated the differential degradation of chloroplasts among the CK, LH, and FQ groups during curing (**Figure 6**). In fresh leaves, the chloroplasts of the CK group exhibited the most rapid disintegration, becoming largely undetectable by 42 °C. Conversely, chloroplasts in the lower-quality leaves (LH and FQ) maintained greater structural integrity at this stage. Ultimately, by the post-curing stage, a complete structural collapse was observed in CK, while the lower-quality leaves still retained relatively intact chloroplasts.

Pre-curing analysis revealed that low-quality leaves possessed markedly fewer and smaller starch granules than CK, with LH displaying the lowest abundance and smallest size. While degradation initiated first in FQ, this group retained substantially more residual starch material after curing than CK. Concurrently, LH exhibited the most sluggish and least complete starch granule degradation. Additionally, the stroma thylakoids in low-quality leaves were more tightly packed than in CK initially, and preserved more residual structures during curing. The grana thylakoids in LH were the most compact, followed by FQ.

**Figure 7:**
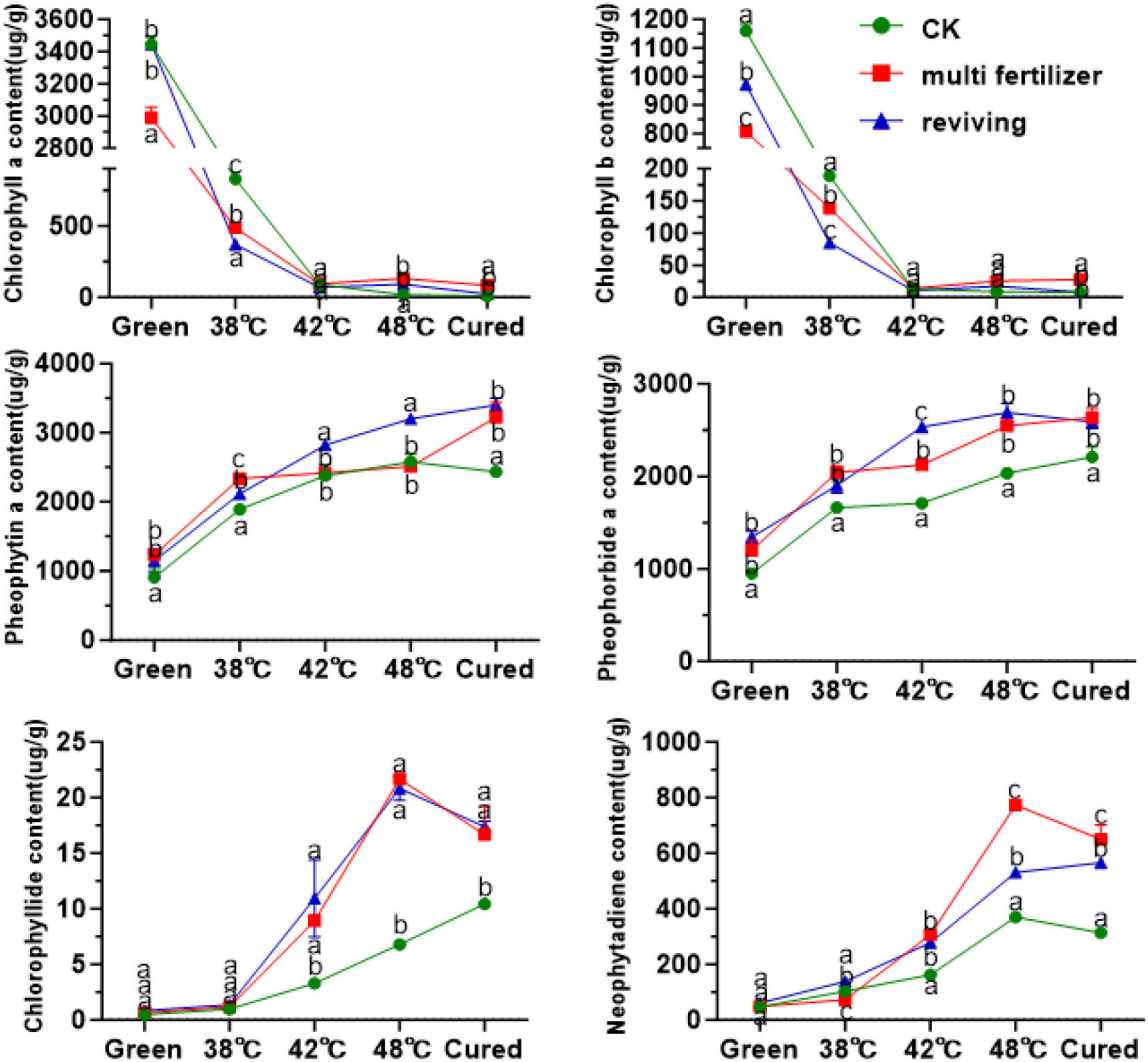
Chlorophyll and its degradation products in tobacco leaves of CK, Multi-fertilizer and Reviving groups during flue-curing.

To elucidate chlorophyll metabolism during flue-curing, we quantified the dynamics of chlorophyll a, chlorophyll b, and their key degradation products, including pheophytin a, pheophorbide a, phytol, and neophytadiene. The results demonstrated a rapid decline in chlorophyll a and b levels across all groups (CK, LH, FQ), which stabilized after the 42 °C stage (**Figure 7**). In line with this degradation, the concentrations of primary catabolites increased significantly. The final degradation rates reached 99.36%, 97.06%, and 99.29% for the CK, LH, and FQ groups, respectively. The chlorophyll a/b ratio shifted from initial values of approximately 3:1, 4:1, and 4:1 in CK, LH, and FQ to final ratios of 1:1, 3:1, and 3:1, respectively. Notably, the accumulation of degradation products was markedly higher in the LH and FQ groups than in the CK group. While the FQ group slightly exceeded the LH group in its content of pheophytin a, pheophorbide a, and phytol, its neophytadiene level was significantly lower.

**Figure 8:**
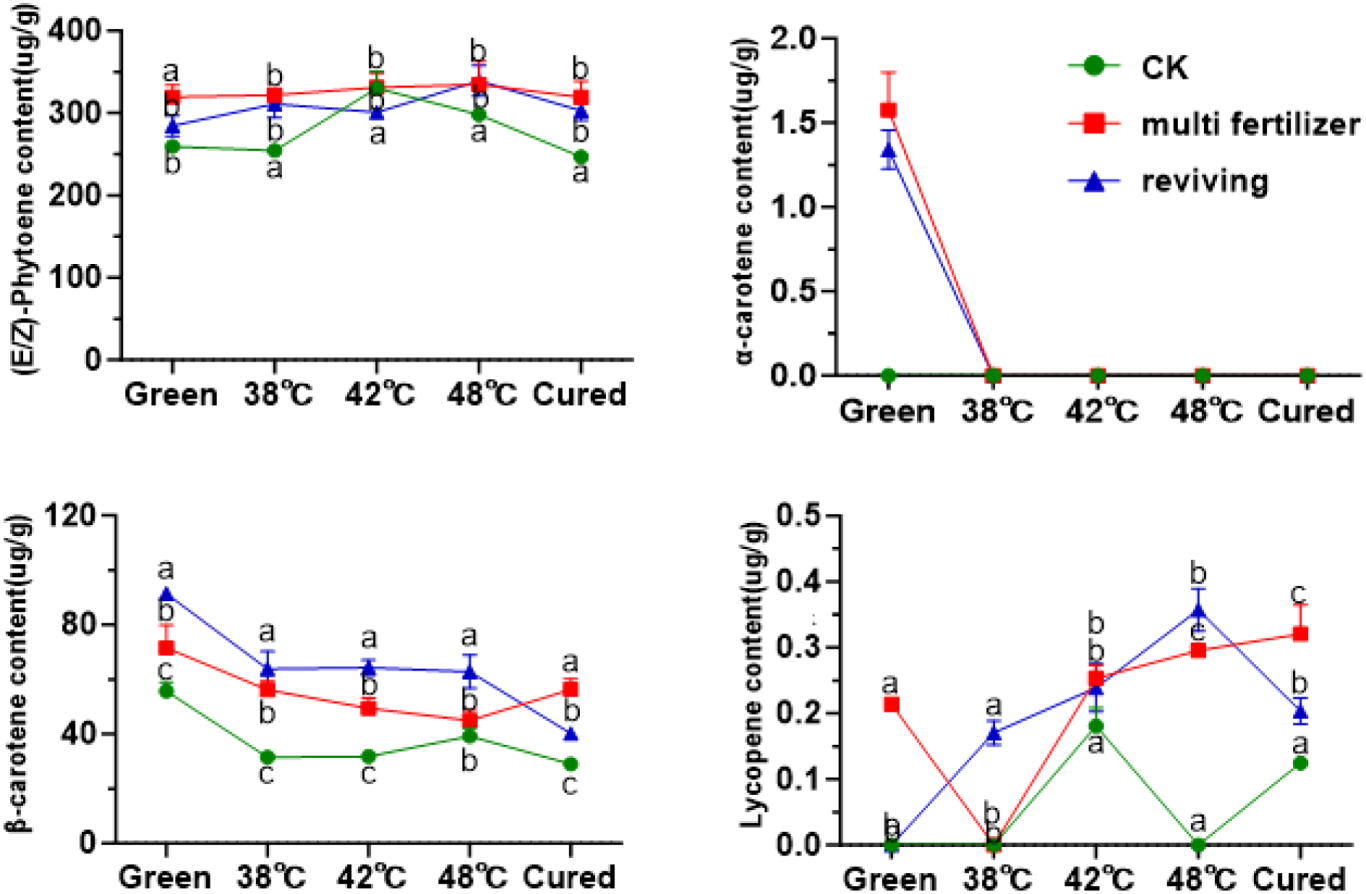
Carotenoid olefins in tobacco leaves of CK, Multi-fertilizer and Reviving groups during flue-curing.

**Figure 9:**
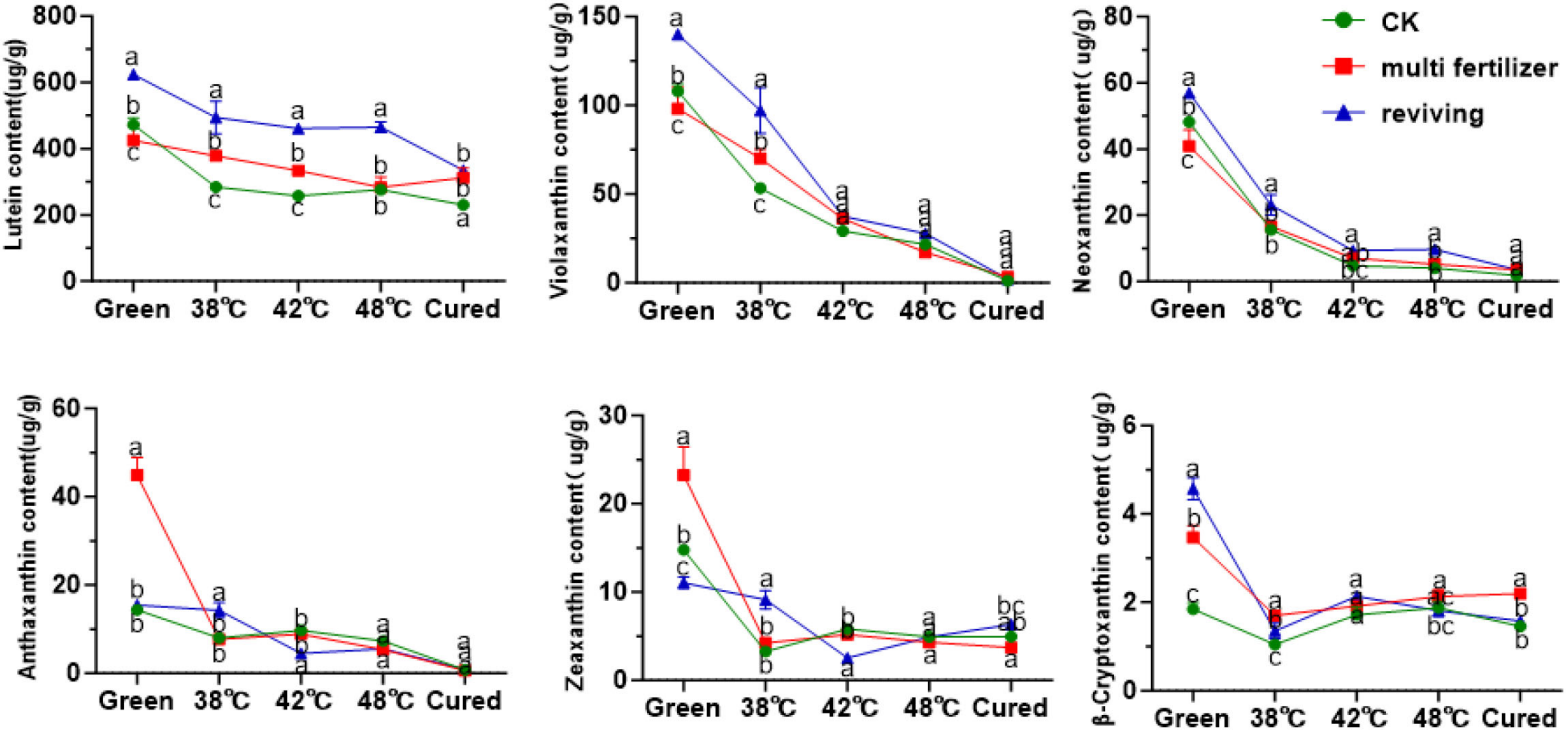
Oxidized carotenoids in tobacco leaves of different qualities during flue-curing.

### 3.6 Carotenoid transformation during the curing of tobacco leaves with different qualities

Beyond chlorophyll metabolism, we also profiled the dynamic changes in carotenoid composition to characterize their behavior in tobacco leaves of varying qualities. Carotenoids were classified into carotenes and oxygen-containing xanthophylls based on their chemical structures. Quantitative analysis demonstrated that lycopene was the predominant carotene in fresh leaves, with its content highest in the CK group and lowest in the FQ group (**Figure 8**). During the curing process, lycopene levels initially increased then decreased, resulting in no statistically significant difference between the initial and final stages. In fresh leaves, β-carotene constituted 17.69%, 18.21%, and 24.23% of the total carotenes in the CK, LH, and FQ groups, respectively. The degradation rates of β-carotene during curing reached 47.94% in CK, 21.21% in LH, and 56.02% in FQ. Notably, the LH group exhibited a significantly lower degradation rate of β-carotene compared to the CK and FQ groups, likely attributable to its distinct physiological characteristics. Lycopene levels remained low throughout the process, with concentrations in some groups falling below the detection limit, and no clear temporal trend was observed. α-Carotene was detectable only in the low-quality leaves prior to curing and became undetectable thereafter, indicating substantial degradation or conversion during the process.

Among oxidized carotenoids, lutein was the predominant component across all three tobacco leaf types (CK, LH, and FQ groups). In fresh leaves, lutein accounted for 71.61%, 66.88%, and 73.24% of total oxidized carotenoids in CK, LH, and FQ groups, respectively (**Figure 9**). During curing, oxidized carotenoid content declined in all leaf types. Degradation rates reached 47.02% in CK group, 43.67% in LH group, and 31.79% in FQ group. Lutein, the primary oxidized carotenoid, degraded by 51.22% in CK group, 26.46% in LH group, and 46.33% in FQ group. Notably, the LH group exhibited significantly lower lutein degradation than CK and FQ groups, highlighting distinct chemical responses to curing among leaf types. Further analysis revealed that the LH and FQ groups contained significantly higher levels of six oxidized carotenoids—lutein, violaxanthin, neoxanthin, antheraxanthin, zeaxanthin, and cryptoxanthin—compared to CK and FQ groups showed the highest concentrations of lutein, violaxanthin, neoxanthin, and cryptoxanthin, whereas the LH group displayed the greatest levels of antheraxanthin and zeaxanthin in fresh leaves.

**Figure 10:**
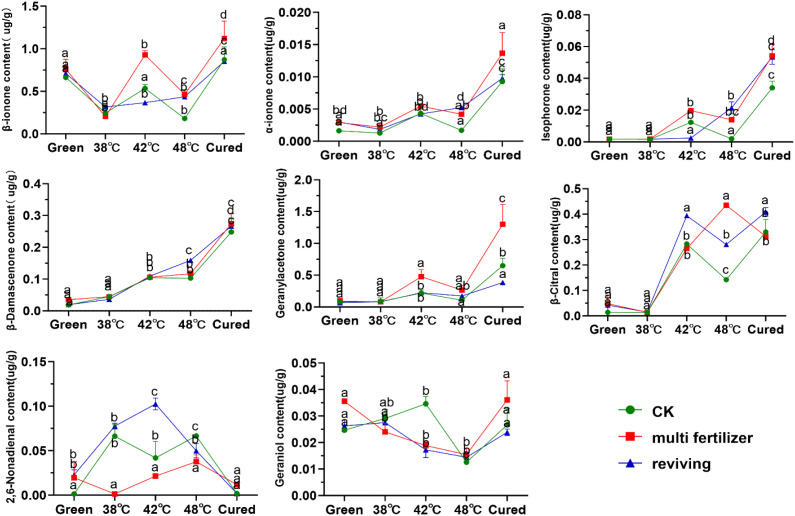
Carotenoid degradation products in tobacco leaves of CK, Multi-fertilizer and Reviving groups during flue-curing.

In this study, we systematically analyzed the primary carotenoid degradation products (e.g., β-ionone, β-damascenone, geranylacetone, isophorone) in tobacco leaves during roasting (**Figure 10**). The degradation product content in CK, LH, and FQ groups exhibited distinct trends: α-ionone, geranylacetone, and isophorone levels generally increased; β-ionone, geraniol, linalool, and α-farnesene showed an initial decline followed by a rise; spirovetivane levels consistently decreased. Further analysis revealed that low-quality leaves (LH and FQ groups) had significantly higher carotenoid degradation product content than CK group. Specifically, the LH group showed the highest levels of β-carotene, α-carotene, isophorone, and geranylacetone, while the FQ group exhibited the most pronounced accumulation of β-damascenone and citral.

## 4 Discussions

### 4.1 Physiological changes in different types of tobacco leaves during curing

During the curing process, significant differences in physiological and structural characteristics were observed among the three types of leaves. The CK group leaves, with optimal maturity, exhibited synchronized color changes and dehydration. Their L-value (lightness) increased steadily, a-value shifted from negative to positive, and b-value (yellowness) rose consistently, resulting in a uniform golden-yellow color. We observed that balanced membrane permeability and efficient water migration supported continuous enzymatic activity, which helped to maintaining equilibrium between pigment degradation and cellular stability (Li et al., 2022). In LH group leaves, dense tissue structure impeded water migration, causing delayed dehydration. Although their strong water retention slowed membrane damage, we observed that incomplete chlorophyll degradation and localized polyphenol oxidation led to uneven dark-yellow patches and delayed browning (Chen et al., 2019). The FQ group leaves, affected by secondary growth, showed metabolic imbalance due to immature cell membranes and high free-water content. During the early curing phase, rapid water loss and a sharp rise in membrane permeability disrupted metabolic activity, which likely led to erratic chlorophyll degradation. This resulted in a mottled yellow-green appearance with grayish discoloration (Yong et al., 2024). The distinct behaviors of these leaves suggest that initial moisture content, membrane stability thresholds, and the spatiotemporal distribution of enzyme activity critically influence the coordination of pigment degradation and water migration during curing, ultimately determining post-curing quality (Yong et al., 2024). However, it is important to note that the analysis regarding the roles of water migration and spatial enzyme distribution provided above is primarily based on correlative physiological data obtained in this study, such as relative electrical conductivity and weight loss patterns. To definitively verify this mechanism, future work would require direct measurements such as water diffusion coefficient assays and enzyme immunolocalization. For instance, applying low-field nuclear magnetic resonance (NMR) to quantify the dynamics of bound versus free water presents a valuable direction for future research.

### 4.2 Carbon and nitrogen metabolism differences in different types of tobacco leaves during curing

Metabolic differences among the three leaf types during curing likely stem from variations in antioxidant regulation and the coordination of carbon-nitrogen metabolism.CK leaves maintained redox balance through stable antioxidant enzyme activity. Efficient starch degradation (via amylase) and regulated nitrogen allocation (via proteases) ensured a dynamic equilibrium between sugar and nitrogen metabolism. In LH leaves, persistently high antioxidant enzyme activity triggered lipid peroxidation, while low amylase activity hindered starch breakdown. Combined with stalled nitrogen metabolism, this created a cycle of oxidative damage and metabolic dysfunction.

FQ leaves showed transient and incomplete antioxidant responses. Despite high amylase activity, low initial starch content led to abnormal post-curing starch and alkaloid accumulation, indicating inefficient metabolic regulation (Changyi et al., 2009). Both excessive and insufficient starch levels disrupt chemical composition in cured leaves, reducing usability (Xiaobin et al., 2019). Chloroplast ultrastructure deteriorated progressively during curing. Intact grana thylakoids, stromal thylakoids, and starch granules in fresh leaves degraded as temperatures rose. Thylakoid structures degraded first, with CK showing the most complete breakdown, while LH and FQ exhibited partial disintegration. Starch granules were nearly fully disrupted at 42 °C, aligning with observed starch degradation patterns. In LH leaves, nitrogen overuse delayed maturity. Compact thylakoid arrangement and insufficient starch accumulation in chloroplasts restricted enzyme-substrate interaction, lowering starch degradation rates compared to CK group. In FQ group leaves, metabolic disruption from secondary growth intensified thylakoid membrane peroxidation, reducing structural stability. Residual starch granules remained significantly higher than in CK (Wang et al., 2012, 2022; Xing-sheng et al., 2024). The key distinction lies in metabolic network integrity. CK leaves achieved efficient starch degradation, pigment conversion, and nitrogen metabolism through synchronized tissue structure and enzyme activity. LH leaves suffered oxidative stress and abnormal chemistry due to physical barriers impeding water migration and enzyme-substrate interaction. FQ leaves exhibited irreversible quality loss from developmental defects, including membrane collapse and metabolic pathway disruption. These findings suggest tailoring curing strategies: prolonging the yellowing phase for LH to enhance enzymatic degradation, and moderating dehydration rates for FQ to restore metabolic continuity, thereby improving overall curing outcomes.

### 4.3 Pigment metabolism differences in tobacco leaves during curing

Lower-quality leaves (LH and FQ groups) exhibited initial levels of chlorophyll degradation intermediates compared to CK during curing. This phenomenon likely originates from delayed leaf maturation induced by nutrient surplus, which subsequently impedes the complete catabolism of these intermediates. Excessive nitrogen application stimulates chloroplast proliferation and attenuates senescence signaling, thereby stabilizing chloroplast membranes and suppressing the activities of chlorophyllase (CHL) and magnesium-dechelatase (MDCase), ultimately constraining chlorophyll degradation (Gaur et al., 2006). We observed a significant accumulation of pheophytin-a in LH leaves. Literature suggests that excess nitrogen can delay senescence-related processes (Mae, 2004).A critical metabolic bottleneck occurs during the conversion of pheophytin-a to phytol via dephytylation, a reaction catalysed by pheophytinase(PPH). We hypothesize that nutrient overload may inhibit PPH activity or disrupt substrate accessibility, thereby arresting this essential process (Heaton and Marangoni, 1996). However, this hypothesized inhibition of PPH (e.g., NtPPH1/2) activity or expression by excess nitrogen remains to be verified by direct measurements, such as qRT-PCR and enzymatic assays in future studies. The abnormal accumulation of chlorophyll metabolites in lower-quality leaves is directly correlated with a deterioration in quality. The photosensitive oxidation of retained pheophytin-a increases susceptibility to browning, while incomplete breakdown due to compromised PPH activity generates unpleasant odours. Reduced carotenoid degradation in these leaves may be due to the suppression of key enzymes by nitrogen. In LH leaves, prolonged excess nitrogen fosters dense cellular architectures that hinder heat and moisture transfer during curing. This consequently diminishes lipoxygenase activity and impedes the enzymatic degradation of lutein and β-carotene. In FQ leaves, chlorophyll re-synthesis during reviving modifies chloroplast ultrastructure, indirectly impairing carotenoid cleavage dioxygenase (CCD) efficacy. This shifts degradation toward non-enzymatic pathways. Under elevated curing temperatures, both LH and FQ leaves favor non-enzymatic oxidative cleavage, which is less efficient than enzymatic conversion, resulting in overall reduced degradation rates (Hua-Dong et al., 2004; Song et al., 2010). Lycopene exhibits particular stability, attributable to its conjugated double-bond system or the absence of specific cleavage enzymes (Popova et al., 2019; Zhonglin et al., 2022). Despite lower degradation rates, the substantially higher initial carotenoid content in LH and FQ leaves still yields greater absolute accumulation of degradation products. For instance, although LH leaves retained 78.79% of β-carotene (degradation rate 21.21%), their elevated baseline enabled considerable product formation. Concurrently, stress-induced shifts in secondary metabolism may further reduce the degradation of neutral aroma compounds, contributing to their apparent accumulation (Wei-Jie and University, 2014).

To substantiate the claim of “aroma deterioration”, we further discuss the sensory implications of these metabolic alterations. The altered pigment catabolite profile—characterized by a relative deficit of key final products like neophytadiene and an accumulation of intermediates such as pheophytin-A—would be predicted to negatively impact the sensory profile. For example, the potent aroma impact of certain carotenoid-derived products like β-ionone means that even minor absolute changes in concentration can have significant sensory consequences. The overall sensory profile likely shifts away from the balanced, pleasant aroma associated with fully degraded products towards one marked by potential off-notes from accumulated intermediates and a lack of characteristic final volatiles (Biao et al., 2006; Erdeng et al., 2018; Shuping et al., 2004; Yaofu et al., 2006).

## 5 Conclusion

This study demonstrates that LH and FQ leaves exhibit inferior visual quality during flue-curing, characterized by uneven yellow-green mottling, dark patches, and grayish discoloration. Instrumental colorimetric analysis confirmed significant quality defects in LH and FQ leaves. Compared to the CK group, both LH and FQ leaves exhibited approximately 10% lower L and b values during the curing process, correlating with the observed dull and uneven coloration, which typically corresponds to lower grades in industrial classification. Tissue stability analysis revealed delayed electrolyte leakage peaks and weight loss in LH leaves compared to CK, whereas FQ leaves underwent these physiological shifts earlier, indicating distinct curing tolerance among different leaf quality types.

Chemically, lower-quality leaves (LH/FQ) displayed elevated pre-curing nitrogen, alkaloid, and protein contents, with significant residual levels post-curing. These were accompanied by dysregulated sugar metabolism and incomplete starch degradation. Enzymatic profiles showed heightened amylase activity in LH/FQ during curing; however, concurrent elevations in antioxidant enzymes and disrupted carbon–nitrogen homeostasis collectively indicate poor curing adaptability.

Ultrastructural examination of chloroplasts in fresh LH/FQ leaves revealed densely stacked thylakoid membranes and reduced starch granules. Their pronounced fragmentation after curing further corroborates the inferior structural resilience of these materials.

Pigment metabolism analyses indicated impaired degradation of both chlorophylls and carotenoids in LH/FQ, accompanied by substantial accumulation of catabolic intermediates. Nitrogen excess likely suppressed key pigment-degrading enzymes while shifting degradation toward stress-induced non-enzymatic pathways. Coupled with higher initial pigment pools and stress-specific pathway activation, this imbalance in degradation products contributes significantly to aroma deterioration in lower-quality leaves.

These findings provide a theoretical basis for optimizing curing protocols for lower-quality leaves. Our results suggest tailored curing strategies: For multi-fertilizer (LH) leaves, extending the yellowing stage at 38 °C (dry-bulb)/36 °C (wet-bulb) by 6–8 hours could promote more complete starch and pigment degradation. For reviving (FQ) leaves, a slower temperature ramp (e.g., 1 °C every 2 hours) from 38 °C to 42 °C is recommended to mitigate the sharp rise in membrane permeability and associated metabolic disruption. Future efforts should focus on modulating curing parameters (e.g., temperature–humidity regimes) and improving agronomic practices (e.g., nitrogen management) to promote more complete pigment degradation and enhance final leaf quality. Furthermore, elucidating the molecular mechanisms governing pigment metabolism could enable targeted strategies for quality enhancement.

## Data Availability

The original contributions presented in the study are included in the article/supplementary material. Further inquiries can be directed to the corresponding authors.
